# Unveiling the complete genome sequence of *Alicyclobacillus acidoterrestris* DSM 3922^T^, a taint-producing strain

**DOI:** 10.1093/g3journal/jkac225

**Published:** 2022-10-14

**Authors:** Inês Carvalho Leonardo, Maria Teresa Barreto Crespo, Frédéric Bustos Gaspar

**Affiliations:** Food & Health Division, iBET, Instituto de Biologia Experimental e Tecnológica, Apartado 12, 2781-901 Oeiras, Portugal; ITQB-NOVA, Instituto de Tecnologia Química e Biológica António Xavier, Universidade Nova de Lisboa, Avenida da República, 2780-157 Oeiras, Portugal; Food & Health Division, iBET, Instituto de Biologia Experimental e Tecnológica, Apartado 12, 2781-901 Oeiras, Portugal; ITQB-NOVA, Instituto de Tecnologia Química e Biológica António Xavier, Universidade Nova de Lisboa, Avenida da República, 2780-157 Oeiras, Portugal; Food & Health Division, iBET, Instituto de Biologia Experimental e Tecnológica, Apartado 12, 2781-901 Oeiras, Portugal; ITQB-NOVA, Instituto de Tecnologia Química e Biológica António Xavier, Universidade Nova de Lisboa, Avenida da República, 2780-157 Oeiras, Portugal

**Keywords:** *Alicyclobacillus acidoterrestris*, spoilage bacteria, taints and off-flavors, complete genome, genome sequencing, assembly, and annotation, function prediction

## Abstract

Several species from the *Alicyclobacillus* genus have received much attention from the food and beverages industries. Their presence has been co-related with spoilage events of acidic food matrices, namely fruit juices and other fruit-based products, the majority attributed to *Alicyclobacillus acidoterrestris*. In this work, a combination of short and long reads enabled the assembly of the complete genome of *A. acidoterrestris* DSM 3922^T^, perfecting the draft genome already available (AURB00000000), and revealing the presence of one chromosome (4,222,202 bp; GC content 52.3%) as well as one plasmid (124,737 bp; GC content 46.6%). From the 4,288 genes identified, 4,004 sequences were attributed to coding sequences with proteins, with more than 80% being functionally annotated. This allowed the identification of metabolic pathways and networks and the interpretation of high-level functions with significant reliability. Furthermore, the additional genes of interest related to spore germination, off-flavor production, namely the *vdc* cluster, and CRISPR arrays, were identified. More importantly, this is the first complete and closed genome sequence for a taint-producing *Alicyclobacillus* species and thus represents a valuable reference for further comparative and functional genomic studies.

## Introduction

During the last decades, different *Alicyclobacillus* (ACB) strains have challenged a variety of commercial activities, including the dairy, bakery, distilling, and beverage industries, since their presence in acidic food matrices, namely fruit juices and other fruit-based products, has been correlated with spoilage events (Merle and Montville 2014). In addition, certain ACB strains have been described as producing metabolites, such as phenolic compounds, with the ability to modify the organoleptic properties of the food matrices they contaminate (Gocmen *et al.* 2005). These off-flavors and odors, described as antiseptic, smoky, and medicinal-like, were shown to be produced by strains belonging to certain ACB species, where *Alicyclobacillus acidoterrestris* is the one associated with the majority of the reported spoilage events (Gocmen *et al.* 2005; Niwa 2005; Danyluk *et al.* 2011; Zhang *et al.* 2013; Roth *et al.* 2021). Thus, *A. acidoterrestris* has been the focus of most studies related to ACB spoilage control by exploring different strategies for the detection and growth inhibition of these bacteria in food matrices (Bahçeci and Acar 2007; Sokołowska *et al.* 2012; Bevilacqua *et al.* 2013; Porębska *et al.* 2017; Hu *et al.* 2020). To continue devising strategies in a targeted way, a thorough characterization of *A. acidoterrestris*, both genetic and phenotypic, is crucial to enhance the knowledge regarding microbial behavior, adaptation, unique features, and, ultimately, control.

Phenotypic traits of the type strain *A. acidoterrestris* DSM 3922^T^ (= ATCC 49025 = LMG 16906) have been widely explored. This gram-positive aerobic bacterium can grow in acidic environments (pH 2.5 – pH 6.0) and at a wide range of temperatures (25°C – 60°C). It can produce subterminal to terminal oval spores, with ω-alicyclic fatty acids in the composition of its membrane, which are distinctive fatty acids present across the majority of ACB species (Wisotzkey *et al.* 1992; Goto *et al.* 2003). This type strain can metabolize various carbon sources and in presence of vanillin, vanillic acid, or other aromatic compounds, it can produce guaiacol, 2,6-dichlorophenol, and 2,6-dibromophenol, which are the compounds identified as the leading cause of consumer complaints of spoiled food products (Silva and Gibbs 2001; Cai *et al.* 2015).

The genome of *A. acidoterrestris* started being extensively explored after 2013 when Shemesh *et al.* (2013) presented a draft genome for the type strain, exclusively obtained from short reads. This genome comprises 207 contigs, where most of the predicted annotations correspond to hypothetical proteins (89.7% of the identified protein-coding sequences). Relevant regions such as the 16S rRNA region and genes involved in guaiacol production, namely the *vdcC* gene, had already been elucidated and used for phylogenetic analysis and the prediction of off-flavor production, respectively (da Costa *et al.* 2009; Wang, Yue, *et al.* 2021).

Despite that, a comprehensive elucidation of the complete *A. acidoterrestris* genomic traits may disclose novel key features with relevance for spoilage control (e.g. spore germination, additional off-flavors producing enzymes) or biotechnological applications (e.g. thermostable enzymes) (Correa-llantén *et al.* 2014; Murphy *et al.* 2020). Bearing this in mind, the aim of this work is to: (1) provide the first complete genome of a taint-producing ACB strain by solving the unassembled draft genome of *A. acidoterrestris* DSM 3922^T^ through sequencing and assembly of long and short reads; (2) functionally annotate most coding sequences, including the ones newly revealed; and (3) present a global functional analysis highlighting distinct features of interest (e.g. off-flavor production-related genes) that may be of great relevance to comparative and functional genomic analysis within this spoilage-associated bacterial group.

## Methods

### Strain origin and growth conditions

*Alicyclobacillus acidoterrestris* DSM 3922^T^, obtained from Deutsche Sammlung von Mikroorganismen und Zellkulturen (DSMZ, Germany), was used in this study. When needed, the strain was cultured either in BAT broth (Scharlau, Spain) or BAT agar (Scharlau, Spain), both adjusted to pH 4.

To obtain adequate bacterial growth to perform the DNA extraction steps, *A. acidoterrestris* DSM 3922^T^ was cultivated from a pure culture grown on BAT agar plates. Isolated colonies were selected and subsequently inoculated in BAT broth. For convenience, the liquid culture was grown for 16 h at 37°C under orbital shaking conditions. A volume of 20 mL of bacterial growth was centrifuged at 4°C, 2,000 *g*, for 10 min, and the pellet was washed twice in 0.9% w/v NaCl (Panreac, USA). Pelleted bacterial cells were resuspended in RNA/DNA Shield reagent (Zymo Research, USA) to obtain an OD_600 nm_ of 11, corresponding approximately to 10^9^ CFU/mL of vegetative bacteria. The bacterial cell suspension was sent to MicrobesNG (http://www.microbesng.com), which performed the DNA extraction, library preparation, and sequencing as described in the sections below.

### DNA extraction

The previously obtained bacterial suspension, 5 – 40 µL, was incubated with 120 µL of Tris-EDTA buffer containing lysozyme and RNase A (ITW Reagents, Barcelona, Spain), both at final concentrations of 0.1 mg/mL, for 25 min at 37°C. Proteinase K (VWR Chemicals, Ohio, USA) and SDS (Sigma-Aldrich, Missouri, USA) were added at final concentrations of 0.1 mg/mL and 0.5% v/v, respectively, and incubated for 5 min at 65°C. Genomic DNA was purified using an equal volume of Solid Phase Reversible Immobilization beads (Beckman Coulter, USA) and finally, resuspended in EB buffer (Qiagen, Germany). DNA was quantified with the Quant-iT dsDNA HS kit (ThermoFisher Scientific, USA) assay in an Eppendorf AF2200 plate reader (Eppendorf UK Ltd, United Kingdom).

### Library preparation and sequencing

Short-read genomic DNA libraries were prepared using the Nextera XT Library Prep Kit (Illumina, San Diego, USA) following the manufacturer’s protocol with modifications: (1) input DNA was doubled and (2) PCR elongation time was increased to 45 s. DNA quantification and library preparation were carried out on a Hamilton Microlab STAR automated liquid handling system (Hamilton Bonaduz AG, Switzerland). The Kapa Biosystems Library Quantification Kit for Illumina (Roche, Switzerland) was used to quantify pooled libraries that were sequenced using the NovaSeq 6000 (Illumina, USA), using a 250 bp paired-end protocol.

Long-read genomic DNA libraries were prepared with the Oxford Nanopore SQK-LSK109 kit with Native Barcoding EXP-NBD196 (ONT, United Kingdom) using 400 – 500 ng of DNA. The barcoded sample was loaded in an FLO-MIN106 (R.9.4.1) flow cell in a GridION system (ONT, United Kingdom).

### Genome assembly

After an initial trimming of Illumina adapters from the totality of raw short reads, the reads were quality and length filtered using Trimmomatic (v0.39) (Bolger *et al.* 2014) with a sliding window of 5, a quality cut-off of Q30, and a length cut-off of 50 bp. The Oxford Nanopore long reads were quality and length filtered using Filtlong (v0.2.1) (https://github.com/rrwick/Filtlong) with a quality cut-off of 90% and length cut-off of 1,000 bp.

*De novo* assembly was performed using Unicycler (v0.4.0) (Wick *et al.* 2017) applying a hybrid assembly with filtered Illumina short reads and Oxford Nanopore long reads. In the same way, Flye (v2.9) (Kolmogorov *et al.* 2019) was also used for *de novo* genome assembly by exclusively using long reads. Results retrieved from both assemblers were evaluated using Geneious Prime 2022.0.1 (https://www.geneious.com), and discrepancies were manually curated by comparing with the obtained reads and the available contigs of the previously published draft genome (Shemesh *et al.* 2013). Bandage (v0.8.1) (Wick *et al.* 2015) was used to visualize the assembly graphs when needed.

### Genome annotation and analysis

The complete assembled genome sequence obtained was submitted to NCBI. The annotation was performed using the NCBI Prokaryotic Genome Annotation Pipeline (PGAP) (version 2021-11-29 build5742), which combines *ab initio* gene prediction algorithms with homology-based methods, enabling an automatic annotation of chromosomes and plasmids (Tatusova *et al.* 2016; Haft *et al.* 2018; Li *et al.* 2021).

The annotated genome was analyzed using different databases to explore genomic features of *A. acidoterrestris* DSM 3922^T^. In this case, BLASTKOALA, KEGG, UniProt, and COG databases were used to characterize individual gene functions, categorize proteins with common functions, and identify different metabolic pathways (Kanehisa *et al.* 2016; Galperin *et al.* 2019; Cantalapiedra *et al.* 2021). In addition, the antiSMASH database was also used (Blin *et al.* 2017) to annotate biosynthetic gene clusters. Furthermore, CGView Server (Grant and Stothard 2008) was used to generate the graphical map of the complete and circular genome, allowing the visualization of sequence features calculated directly from the primary sequence (coding sequences, GC content, GC skew, RNA sequences, and repeat regions).

## Results and discussion

### *De novo* complete genome assembly and corroboration

**Fig. 1.**
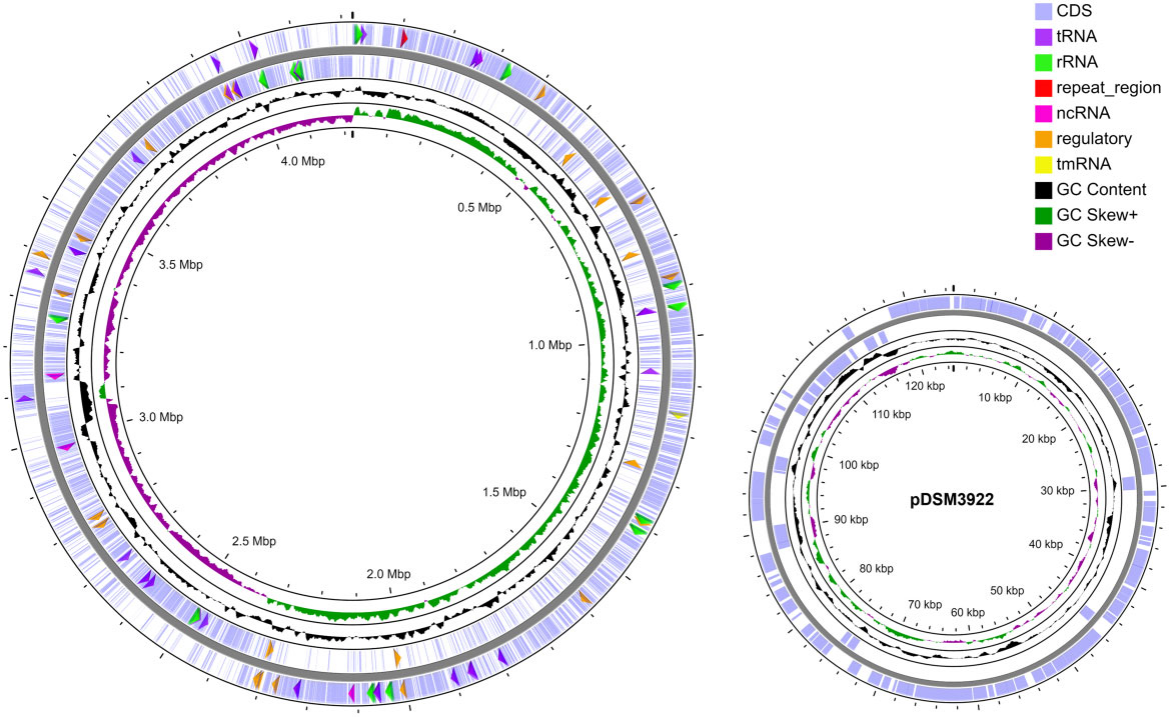
Graphical circular maps of *A. acidoterrestris* DSM 3922^T^ chromosome and its plasmid pDSM3922. From the outside to the inside: coding sequencing (CDS) regions identified on the forward strand, CDSs identified on the reverse strand, GC content , and GC skew. All genomic features identified, namely RNA genes (tRNA, rRNA, tmRNA, and ncRNA), regulatory elements, and repeat regions associated with CRISPR arrays, on the forward and reverse strands (except CDSs) were labeled with clockwise and counterclockwise arrows, respectively.

In this work, Illumina and Oxford Nanopore were the platforms selected to obtain sequencing data to perform a hybrid assembly of short and long reads and obtain the complete genome sequence of *A. acidoterrestris* DSM 3922^T^. This combination of both reads was previously shown to display promising and reliable results regarding the complete assembly of complex bacterial genomes (De Maio *et al.* 2019). Long reads, obtained from the Oxford Nanopore platform, provide the required scaffolding and genome structure information. The addition of short reads, retrieved from the Illumina platform, allows a detailed assembly at the local scale, correcting the inherently error-prone long reads (Ruan *et al.* 2020). The integration of both quality and length filtered datasets (Supplementary Table 1) enabled the reliable, complete, and high coverage sequencing of *A. acidoterrestris* DSM 3922^T^ genome, with short reads (50 – 251 bp) displaying an average genomic coverage of 70× that supplemented the average coverage of 36× obtained from long reads (1,000 – 119,497 bp). As a result, the complete genome of this strain was revealed to be composed of one long chromosome with 4,222,202 bp and one plasmid with 124,737 bp (Fig. 1, Table 1).

**Table 1.** Genomic features of *A. acidoterrestris* DSM 3922^T^.

Attribute	Draft genome (AURB00000000)					Complete genome		
	Original contigs	Reannotated contigs	Reannotated and merged contigs					
			Total	Contigs that map to		Total	Chromosome (CP080467)	pDSM3922 (CP080468)
				Chromosome	pDSM3922			
N° of contigs	207	207	175	166	9	2	1	1
Genome size (bp)	4,063,548	4,063,548	4,062,183	3,941,656	120,527	4,346,939	4,222,202	124,737
G + C content (%)	52.2	52.2	52.2	52.4	46.5	52.1	52.3	46.6
Total genes	4,240	4,087	4,077	3,964	113	4,288	4,174	114
Pseudogenes	4	87	89	89	–	109	105	4
Total CDSs	4,103	3,949	3,939	3,826	113	4,113	3,999	114
CDSs with protein	3,883	3,862	3,850	3,737	113	4,004	3,894	110
Hypothetical proteins	3,483	690	682	629	53	678	627	51
rRNAs	2	2	2	2	–	39	13–13–13 (5S–16S–23S)	–
ncRNAs	4	4	4	4	–	4	4	–
tmRNAs	1	1	1	1	–	1	1	–
Regulatory	–	23	23	23	–	38	38	–
tRNA	131	131	131	131	–	131	131	–
CRISPR arrays	1	1	1	1	–	1	1	–
Transposases	15	71	71	63	8	203	191	12

Annotations were obtained using PGAP version 2021-11-29 build5742 (GenBank accession number indicated between brackets).

The *de novo* assembled genome sequence of *A. acidoterrestris* DSM 3922^T^ was analyzed against the previously available draft genome (Shemesh *et al.* 2013) to corroborate the nucleotide assembly obtained and evaluate the knowledge increment provided by this study. For that, the contigs from the draft genome were mapped to the *de novo* assembly using Minimap2 (Li, 2018). The mapped draft contigs that overlapped were merged and manually trimmed, which reduced the number of draft contigs from the initial 207 to 175 (Table 1, Supplementary Table 2). This work describes a plasmid in the *A. acidoterrestris* DSM 3922^T^ genome for the first time. However, sequences that belong to plasmid pDSM3922 were already present in the draft genome. A total of 16 contigs from the draft genome, which could be further merged into 9 contigs (Table 1, Supplementary Table 2), mapped to this plasmid. Nonetheless, over 4 kbp from the plasmid are missing in the draft genome, which previously hindered its circularization and completion, unlike what is presented in this study. The remaining 166 merged contigs (originating from 191 contigs) mapped to the chromosome, revealing that more than 280 kbp were missing in the draft genome (Table 1, Supplementary Table 2). The hybrid assembly strategy performed in this work enabled not only the reconstitution of 2 circular genomic sequences (chromosome and plasmid) but also the addition of nucleotide regions that had not previously been elucidated in the draft genome and that are now fully accessible.

Any occasional nucleotide divergences observed between the merged contigs from the draft genome and the complete *A. acidoterrestris* DSM 3922^T^ genome were corroborated by the high coverage provided by the short reads obtained. Therefore, the mapping, comparison, and corroboration performed fully sustain the structure and the sequence of the obtained assembly. These findings support that the application of long-read sequencing platforms can solve unassembled regions when using Illumina-only assemblies (Wick *et al.* 2017). Nonetheless, the manual curation of assembly results retrieved from different sequencing and assembly strategies should still be considered a practical and advantageous approach due to the challenges that next-generation sequencing platforms still encounter and the variety of available pipelines with alternative assembly algorithms (e.g. Unicyler, Flye, SPAdes) (Bankevich *et al.* 2012; De Maio *et al.* 2019).

### Genome annotation

The annotation of the *de novo* assembled genome sequence of *A. acidoterrestris* DSM 3922^T^ was performed with the PGAP pipeline (Tatusova *et al.* 2016) (Table 1). In this complete genome, a total of 4,288 genes were identified, from which 4,113 were recognized as coding sequences (CDSs) and 4,004 of them codifying for proteins. Besides the short- and long-read sequencings supporting the existence of a plasmid, the genome annotation confirms it. Homologs of *rep* and *par* genes, required for rolling circle plasmid replication and the control of plasmid partition, respectively (Thomas *et al.* 2017), were identified in plasmid pDSM3922, with genes *parA*, *parB*, *parM*, and a sequence that codes for a replication-relaxation family of proteins, being consequently annotated.

To thoroughly and consistently compare the annotations from the complete genome, all 207 contigs of the draft genome, as well as the 175 newly merged contigs, a reannotation of all contigs was performed with the same PGAP pipeline (Table 1). Regarding the draft genome, the reannotation updated the number of genes from 4,240 to 4,087 and the number of CDSs from 4,103 to 3,949 CDSs. With the merging of contigs, and the removal of overlapping sequences, the annotations were further updated, and the number of genes and CDSs reduced to 4,077 and 3,939, respectively. Moreover, a significant reduction (∼80%) of the amount of CDSs annotated as hypothetical proteins were observed between the draft genome contigs and the complete genome sequence, as it was now possible to functionally annotate the majority of sequences (3,312 functionally annotated CDSs with protein out of a total of 4,004, where 678 remain hypothetical proteins), considering the Protein Family Model collection (Tatusova *et al.* 2016).

The mapping of the merged contigs to the complete genome revealed that the gaps that remain, extending in length between 25 and 7,469 bp in both chromosome and plasmid sequences, mainly contain transposase/integrase sequences (Supplementary Table 3). The high abundance of mobile genetic elements present along the complete genome (224 CDSs including transposases, integrases, and recombinases) suggests an increased plasticity and adaptability, similar to what was previously observed for the *Alicyclobacillus acidocaldarius* DSM 466^T^ type strain (Mavromatis *et al.* 2010). These remaining gaps also accommodate additional genes that were absent in the draft genome contigs, including genes related to spore germination (e.g. spore germination protein Ger(x)C), genome replication (e.g. DNA polymerase IV), or gene expression regulation (e.g. helix-turn-helix domain-containing proteins). In addition, 12 repetitions of rRNA genes (Supplementary Table 3) were also identified, which can be relevant for phylogenetic analysis as it was previously demonstrated that variations in multiple copies of the 16S rRNA genes of bacteria could support a proper species identification, namely within the Firmicutes phylum (Ibal *et al.* 2019).

**Fig. 2.**
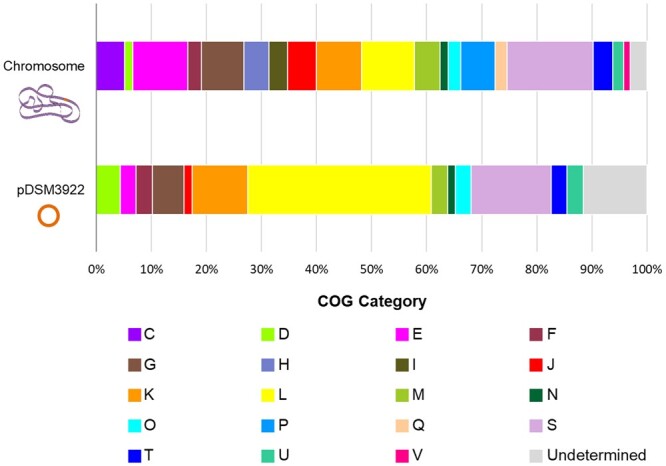
Classification of the clusters of orthologous groups of proteins (COGs) by functional categories of *A. acidoterrestris* DSM 3922^T^. Data retrieved from eggNOG-mapper (Huerta-Cepas *et al.* 2019; Cantalapiedra *et al.* 2021) corresponding to the analysis of the chromosome and plasmid pDSM3922. One-letter abbreviations were used for the functional categories: **C**, energy production and conversion; **D**, cell division and chromosome partitioning; **E**, amino acid metabolism and transport; **F**, nucleotide metabolism and transport; **G**, carbohydrate metabolism and transport; **H**, coenzyme metabolism; **I**, lipid metabolism; **J**, translation, including ribosome structure and biogenesis; **K**, transcription; **L**, replication, recombination and repair; **M**, cell wall structure and biogenesis and outer membrane; **N**, secretion, motility and chemotaxis; **O**, molecular chaperones and related functions; **P**, inorganic ion transport and metabolism; **Q**, secondary metabolites biosynthesis, transport, and catabolism; **S**, function unknown; **T**, signal transduction; **U**, intracellular trafficking, secretion, and vesicular transport; **V**, defence mechanisms.

Further functional analyses of the predicted 4,004 CDSs with protein enabled the attribution of KEGG orthology (KO) identifiers (Kanehisa *et al.* 2016) to about 78% of sequences, and the classification of 86% of sequences within functional categories of Clusters of Orthologous Groups of proteins (COGs) (Supplementary Tables 4–6) (Galperin *et al.* 2019). KEGG annotation server Blast KEGG Orthology And Links Annotation (BlastKOALA) enabled the identification of KO identifiers co-related with all the 22 functional categories defined on the database, including energy and lipid metabolism, environmental information processing, metabolism of cofactors and vitamins, and metabolism of terpenoids and polyketides (Supplementary Table 5) (Kanehisa *et al.* 2016). As expected, most annotated proteins were correlated with essential cellular functions such as genetic information processing, signaling and cellular processes, and carbohydrate and amino acid metabolism (Supplementary Table 5). Using the COG database, it was possible to identify protein-coding sequences from 19 different functional categories (Fig. 2, Supplementary Table 6), covering all COG classes: (1) cellular processes and signaling, (2) information storage and processing, (3) metabolism, and (4) poorly characterized. Categories correlated to essential functions as genome replication, recombination, and repair (L), transcription (K), translation, including ribosome structure and biogenesis (J), amino acid transport and metabolism (I), and carbohydrate metabolism and transport (G) were predominant, corresponding to ∼60% of identified proteins, which was expected considering previous studies (Konstantinidis and Tiedje 2004; Datta *et al.* 2020). Other relevant functional categories, including protein sequences related to the *A. acidoterrestris* DSM 3922^T^ ability to resist extreme environments (e.g. multidrug resistance efflux pumps, type II secretion system proteins), were also identified. Coding sequences involved in sporulation and germination mechanisms, which are key processes for ACB stress resistance ability, were further explored. In this work, over 70 different sequences encoding for germination and spores’ production-related proteins were annotated (Supplementary Table 6). These sequences include *cotJ* operon proteins (CotJA and CotJB), required for the normal formation of the inner layers of the spores’ coat (Henriques *et al.* 1995), a variety of Spo proteins, correlated with all 5 key stages of sporulation, and transcriptional regulators as GerE, that direct the transcription of several genes (e.g. *cotX*) responsible for structural components of the protein coat encasing mature spore (Riley *et al.* 2020).

The genetic information contained in the plasmid could play an essential role in *A. acidoterrestris* DSM 3922^T^ adaptation to different environments. Several transcription regulators were annotated, namely, the transition state regulator AbrB previously described in *Bacillus* spp. as a key factor in cell survival by regulating spore formation, competence, and biofilm development (Sullivan *et al.* 2008). In addition, proteins involved in SOS response and DNA repair, type II secretion system proteins, and various transporters were annotated in pDSM3922 (F and U functional categories) and identified as features of interest for cell resistance. Genes related to sporulation and germination processes that were found in the chromosome were also identified in the plasmid sequence. Two spore germination proteins, one Ger(x)C family protein and a transition-state regulator (AbrB/MazE/SpoVT family of DNA-binding domain-containing proteins) were annotated, suggesting that *A. acidoterrestris* DSM 3922^T^ plasmid may benefit these spore-forming bacteria during different physiological stages, probably through compensation or increase in proteins translated from the chromosome (Eijlander *et al.* 2016).

These results support that a detailed analysis of COG categories allows not only a detailed functional annotation of sequenced genomes but can also give invaluable information for further comparative studies of related organisms (e.g. prediction of novel functional systems and alternative forms of enzymes, identification of missing and/or undetected genes, comparison of organisms by COG functional categories) (Galperin *et al.* 2019).

### Taints and off-flavors production

Taint-producing ACB isolates, like *A. acidoterrestris* DSM 3922^T^, are known for producing different phenolic compounds, namely guaiacol, 2,6-dichlorophenol, and 2,6-dibromophenol, that can lead to the spoilage of food products (Siegmund and Pöllinger-Zierler 2006; Sokołowska *et al.* 2013). Most studies related to the production of these off-flavors highlight guaiacol production, from either vanillin or vanillic acid, as the most significant spoilage concern. This compound has been identified as the most frequently produced off-flavor by ACB isolates and is usually present at higher concentrations than either halophenols (Gocmen *et al.* 2005). The metabolic pathways leading to guaiacol production have already been thoroughly described. Vanillin is first oxidized to vanillic acid by a vanillin dehydrogenase (Vdh) and then nonoxidatively decarboxylated to guaiacol by a vanillic acid decarboxylase (Cai *et al.* 2015; Gallage and Møller 2015). Recently, Wang, Liang, *et al.* (2021) presented transcriptomic and proteomic data from a different strain, *A. acidoterrestris* DSM 3923, which supports this mechanism of guaiacol production.

This work made possible the identification in the complete genome sequence of *A. acidoterrestris* DSM 3922^T^ of several key genetic traits from the guaiacol production metabolic pathway, adding valuable information to the already available draft genome annotations. The first step in that conversion, when starting from vanillin, is catalyzed by the enzyme Vdh (Gallage and Møller 2015). The vanillin dehydrogenase coding gene has not been found in *A. acidoterrestris* genomes, but a putative *vdh* was recently proposed considering protein sequence identity with *Bacillus subtilis* strain 168 *vdhT* (sequence NP_388616.1) (Borriss *et al.* 2018; Luong *et al.* 2021).

In this work, all 9 annotated coding sequences belonging to the aldehyde dehydrogenase family of proteins were further explored to evaluate which could be responsible for the conversion of vanillin to vanillic acid. For that, protein sequences were analyzed with BLASTp (McGinnis and Madden 2004), to search for closely related homologs from the same phylum (Firmicutes) but belonging to different genera (Supplementary Table 7). To assist the prediction of proteins relevant for guaiacol production, and considering the previous study of Luong *et al.* (2021), vanillin dehydrogenase of *B. subtilis* 168 strain and other highly similar proteins (>99.6% pairwise identity; *E*-value = 0) were aligned to all 9 *A. acidoterrestris* DSM 3922^T^ aldehyde dehydrogenases. Multiple sequence alignment using the MAFFT program (Katoh and Standley 2013) suggested that the aldehyde dehydrogenase annotated with the locus tag K1I37_11200 of *A. acidoterrestris* genome can play a relevant role in vanillin conversion to guaiacol, being responsible for an intermediate conversion to vanillic acid, by presenting the highest identity (48%) with other known vanillin dehydrogenases (Supplementary Table 8).

**Fig. 3.**
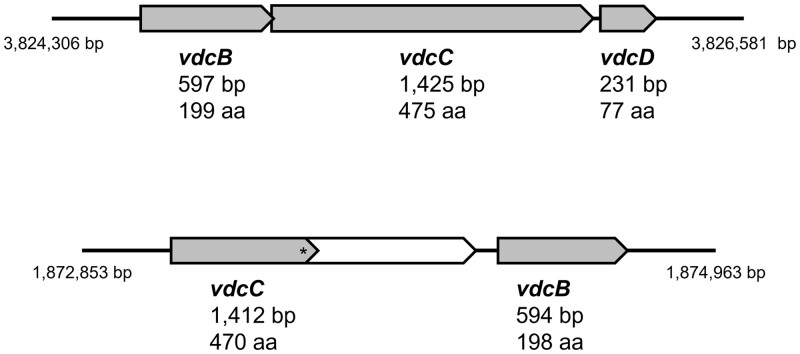
Vanillic acid decarboxylase-related genes annotated in the chromosome of *A. acidoterrestris* DSM 3922^T^: a) Complete *vdc* gene cluster; b) Incomplete *vdc* gene cluster, where “*” indicates the presence of a frameshift mutation.

The following conversion step, catalyzed by a vanillic acid decarboxylase, has been previously described as being coded by the *vdcBCD* cluster (Luong *et al.* 2021). In the draft genome, the *vdcC* gene, coding for subunit C of this decarboxylase, was already annotated in the forward strand of contig AURB01000113. However, the complete information of the genes coding for the remaining subunits (*vdcB* and *vdcD*) from this cluster was lacking and identified as hypothetical proteins. In this work, *vdcB* and *vdcC* were successfully identified by PGAP. The automatically attributed gene names were manually adjusted from the *ubi* family of genes (*ubiX* and *ubiD*) to the *vdc* family of genes (*vdcB* and *vdcC*, respectively) for consistency purposes with the most common nomenclature within annotated nucleotide sequences from ACB species (Álvarez-Rodríguez *et al.* 2003; Gulmezian *et al.* 2007; Dekowska *et al.* 2018; Połaska *et al.* 2021), therefore facilitating comparisons in future studies. Regarding *vdcD*, the annotation was confirmed by sequence identity with another *vdc* cluster sequence already available (accession number KX453673) (Dekowska *et al.* 2018) and added manually. Complementing what was previously described in the draft genome, a complete *vdc* gene cluster is now annotated in the forward strand of *A. acidoterrestris* DSM 3922^T^ chromosome between positions 3,824,306 and 3,826,581 bp. It is composed of *vdcB* with 597 bp (199 aa), *vdcC* with 1,425 bp (475 aa), and *vdcD* with 231 bp (77 aa) (Fig. 3). Interestingly, homologs of *vdcB* (59% identity) and *vdcC* (41% identity) were found in another region of the chromosome, in the forward strand between 1,872,853 and 1,874,963 bp (Fig. 3). However, the *vdcD* gene is missing, this *vdcC* gene has a frameshift, and both genes found (*vdcB* and *vdcC*) are not in the same order as the proposed *vdcBCD* cluster, which questions the function of these homologs in contributing to guaiacol production and acting as an actual cluster (Fig. 3).

Besides the complete genome of *A. acidoterrestris* DSM 3922^T^ described here, only 2 other completely sequenced reference genomes from verified *Alicyclobacillus* species are publicly represented on the Genome resource page at NCBI. The 2 available reference genomes, *A. acidocaldarius* DSM 446 (Accession: GCF_000024285.1) (Mavromatis *et al.* 2010) and *Alicyclobacillus mengaziensis* S30H14 (Accession: GCF_017298635.1) (Jiang *et al.* 2022), were used to compare the taint production potential of these strains. Unsurprisingly, similar nucleotide sequences to the *vdc* gene cluster were not found in the *A. acidocaldarius* genome sequence, as this isolate is not described as a guaiacol producer (Van Luong *et al.* 2019). Currently, there is no description of the ability of *A. mengaziensis* to produce guaiacol (Jiang *et al.* 2022), but the absence of nucleotide sequences in the complete genome with similarity to genes from the *vdc* cluster suggests that this isolate is not a producer of that off-flavor either.

In the transcriptomic and proteomic data published by Wang, Liang, *et al.* (2021), additional genes with altered expression levels were identified as related to guaiacol production. The information provided by that work enabled the annotation of sequences surrounding the *vdc* gene cluster, previously identified as hypothetical proteins (Shemesh *et al.* 2013). These coding sequences have now their products identified and annotated in the complete genome. They include, downstream of the *vdc* gene cluster, a pyridoxamine 5′-phosphate oxidase family protein (locus tag K1I37_18815), identified as the second most highly upregulated gene during guaiacol production, and, upstream of the *vdc* gene cluster, the transcriptional regulator LysR (locus tag K1I37_18795), described as positively regulating the *vdc* gene cluster. In addition, the benzoate/H(+) symporter BenE (locus tag K1I37_18790), involved in guaiacol excretion from cells (Wang, Liang, *et al.* 2021), has also been identified. These sequences were compared with the hypothetical proteins previously annotated in the draft genome and only the benzoate/H(+) symporter BenE coding sequence was adjusted in size from 1,260 to 1,254 bp, losing 2 amino acids at the N-terminus (420 aa to 418 aa).

Lastly, no homologs of the genes involved in the pathways leading to vanillin production, from either ferulic acid or vanillyl alcohol (Nguyen *et al.* 2011; Graf *et al.* 2016), were identified in the genome. BLAST searches using phenolic acid decarboxylase (BSU_34400) (Borriss *et al.* 2018) and vanillyl-alcohol oxidase (PDIP_52730) (Marcet-Houben *et al.* 2012) reference sequences, responsible for the conversion of ferulic acid and vanillyl alcohol to vanillin, respectively, were absent from *A. acidoterrestris* DSM 3922^T^ genome sequence.

In addition, protein-coding sequences related to the production of halophenols were also explored. It was possible to identify 13 sequences annotated in *A. acidoterrestris* DSM 3922^T^ chromosome as alpha/beta hydrolases, 12 more than in the draft genome. Some might contribute to the degradation of aromatic compounds when considering their alignment with other hydrolases from *Bacillus* spp. (e.g. 51% identity with the putative hydrolase MhqD from *B. subtilis* strain 168, sequence NP_389837.1), but their specificity as bromoperoxidases is uncertain (Borriss *et al.* 2018). Compared with the extensive information available on guaiacol production by ACB isolates, it is evident that the mechanisms behind halophenols’ production are lacking and should be further explored. The availability of the complete genome sequence of *A. acidoterrestris* DSM 3922^T^ described here can be helpful to advance the functional analysis of off-flavor production.

### Biotechnological applications

The complete genome annotation performed in this work also provides information regarding *A. acidoterrestris* genomic features that might be of interest to medicine, food, and biotechnological applications. Enzymes that are currently relevant for distinct markets, such as pharmaceuticals, detergents and textiles, food and beverages, biofuels, and chemicals (Adrio and Demain 2014), were searched along *A. acidoterrestris* DSM 3922^T^ genome. Coding sequences with promising protein products including 4 lipases, 22 esterases, 101 hydrolases, 4 peroxidases, 28 oxidases, and 267 transferases were identified. A superoxide dismutase (locus tag K1I37_07655) that might have the potential to be used as a food additive, similarly to the one produced by *A. acidocaldarius* previously studied by Dong *et al.* (2021), was also identified.

In the last decades, several enzymes from ACB species have been explored due to their increased stability and activity at different temperatures and pH values when compared to other commonly used enzymes. *A. acidocaldarius*, for instance, has been previously used as a thermoacidophilic model organism for molecular and biochemical studies of its enzymes (Di Lauro *et al*. 2006), and *Alicyclobacillus acidiphilus* was already investigated regarding its β-glucosidase potential to hydrolyze glucovanillin to vanillin and other flavor compounds commonly found in vanilla (Delgado *et al*. 2021), conserving its activity at higher operating temperatures. Acidophilic enzymes, such as the ones produced by ACB species, have numerous applications, particularly in the production of sugar syrups from starch, and bioremediation (Mesbah 2022). Undoubtedly, exploring all these extremophile-derived enzymes can reveal different alternatives for a broad range of medical, food, and biotechnological applications, and functional annotations like the one described in this work can provide valuable information so promising sequences can be identified, *de novo* synthesized, codon-optimized, cloned, and heterologously expressed to reach large scale production (Karan *et al.* 2012; Littlechild 2015; Mesbah 2022).

The presence of biosynthetic gene clusters was investigated using the antiSMASH database (Blin *et al.* 2017). This database enables the identification of gene clusters involved in secondary metabolite production, known as the primary source of bioactive compounds with interest for medical or biotechnological applications (Blin *et al.* 2017). As expected, and considering previously identified biosynthetic gene clusters in distinct ACB and *Bacillus* species (Hamdache *et al.* 2011; Ideno *et al.* 2018; Wang, Liang, *et al.* 2021), 16 regions of interest were identified in the chromosome of *A. acidoterrestris* DSM 3922^T^ that are involved in the production of saccharides, fatty acids, terpenes, and beta-lactones (Supplementary Table 9). These identified clusters may be explored to efficiently produce metabolites relevant either for biotechnological, cosmetic, or medicinal applications (e.g. antioxidant and anti-inflammatory properties of terpenes) (Helfrich *et al.* 2019; Del Prado-Audelo *et al.* 2021; Catinella *et al.* 2022).

In addition, a repeated region associated with the CRISPR/Cas (clustered regularly interspaced short palindromic repeats and CRISPR-associated protein) system was also identified in the complete genome sequence of *A. acidoterrestris* DSM 3922^T^. This CRISPR array is preceded by 3 different Cas enzymes (Cas2, Cas1, and Cas12b) and can be found in other ACB species (Mavromatis *et al.* 2010). These enzymes have been explored as promising alternatives to CRISPR/Cas9 systems since they are derived from thermoacidophilic bacteria and can maintain their nuclease activity under extreme conditions. This increased thermal stability and robustness led different authors to explore ACB-derived Cas enzymes for plant genome editing, mammalian genome editing in human and mouse cells, and viral genome detection, thus presenting valuable features for different promising medical and biotechnological applications (Teng *et al.* 2018; Ali *et al.* 2020; Ming *et al.* 2020).

### Safety-related features

Despite the ability of different ACB isolates to cause serious spoilage issues on different food matrices, no health and safety concerns have yet been related. In addition, previous studies, where *A. acidoterrestris* spores were directly injected into mice or spore-inoculated juices were fed to guinea pigs, revealed no pathogenic potential in the animal models tested, with no death or illness symptoms observed (Walls and Chuyate 2000). Nevertheless, genomic features of *A. acidoterrestris* DSM 3922^T^ that could be linked to health risks or impact food safety were assessed in this study. Antibiotic resistance determinants and acquired resistance genes were explored using ResFinder, ResFinderFG, and KmerResistance databases (Clausen *et al.* 2016; Bortolaia *et al.* 2020). ResFinder and ResFinderFG analyses were performed testing all available parameters, including the minimum percentages for length and identity thresholds (20% and 30%, respectively), without identifying any acquired antibiotic resistance genes or determinants. Similarly, no acquired antibiotic resistance genes were identified using the KmerResistance database with different scoring methods, host databases, and minimum available percentages for identity and depth correlation thresholds (10% and 5%, respectively). Furthermore, the absence of any virulence genes related to toxin production was confirmed using all the available data from different species on the VirulenceFinder database (Tetzschner *et al.* 2020). These results support the characterization of *A. acidoterrestris* DSM 3922^T^ as nonpathogenic bacteria that may cause issues in the quality of food products but are not a food safety hazard (Pornpukdeewattana *et al.* 2020).

## Supplementary Material

jkac225_Supplemental_Material_Tables_S1_S2_S3_S4_and_S9

jkac225_Supplementary_Material_Tables_S5_S6_S7_and_S8

## Data Availability

The data underlying this article are available in GenBank of NCBI at https://www.ncbi.nlm.nih.gov and can be accessed with the accession numbers CP080467 and CP080468, assigned to the sequences of *A. acidoterrestris* DSM 3922^T^ chromosome and plasmid pDSM3922, respectively. The associated BioProject, BioSample, and SRA numbers are PRJNA751022, SAMN20503121, and SRR17245873 for long reads and SRR17245874 for short reads, respectively. Supplemental material is available at G3 online.
