# Supplementary material for: Unveiling the complete genome sequence of Alicyclobacillus acidoterrestris DSM 3922T, a taint-producing strain
Source: G3 (Bethesda). 2022 Oct 14;12(12):jkac225. doi: 10.1093/g3journal/jkac225 (PMC9713406; doi:10.1093/g3journal/jkac225)
Supplement: jkac225_Supplemental_Material_Tables_S1_S2_S3_S4_and_S9 [file jkac225_supplemental_material_tables_s1_s2_s3_s4_and_s9.docx]

**Page 2**

**Table S1 – Quantity, size and, quality of reads retrieved from Oxford Nanopore (long reads) and Illumina (short reads) sequencing platforms.**

**Page 3**

**Table S2 – Draft contigs that mapped to the complete genome sequence of *A. acidoterrestris* DSM 3922^T^.**

**Page 7**

**Table S3 – Sequence annotations of former gaps present in the draft genome of *A. acidoterrestris* DSM 3922^T^.**

**Page 8**

**Table S4 – Number of coding sequences with protein in of *A. acidoterrestris* DSM 3922^T^ complete genome that were functionally annotated using KEGG and COG databases.**

**Page 9**

**Table S9 - Biosynthetic gene clusters identified in the complete genome of *A. acidoterrestris* DSM 3922^T^ using antiSMASH database.**

**Table S1 – Quantity, size and, quality of reads retrieved from Oxford Nanopore (long reads) and Illumina (short reads) sequencing platforms.**

**Long reads**

|  | Number of sequences | Min. length | Mean length | Max. length | Total bases | At least Q20 | At least Q30 |
| --- | --- | --- | --- | --- | --- | --- | --- |
| SRR17245873 | 48 202 | 14 | 3 311 | 119 497 | 159 582 043 | 59.8% | 26.9% |
| Filtered reads | 37 705 | 1 000 | 4 062 | 119 497 | 153 157 977 | 59.9% | 27.0% |

**Short reads**

|  | Paired end | Number of sequences | Min. length | Mean length | Max. length | Total bases | At least Q20 | At least Q30 |
| --- | --- | --- | --- | --- | --- | --- | --- | --- |
| SRR17245874 | 1 | 1 052 099 | 36 | 245 | 251 | 257 630 132 | 97.1% | 91.1 |
|  | 2 | 1 052 099 | 36 | 241 | 251 | 253 804 553 | 96.9% | 91.0 |
| Filtered reads | 1 | 771 675 | 50 | 199 | 251 | 153 702 775 | 99.5% | 96.5% |
|  | 2 | 771 675 | 50 | 197 | 251 | 152 198 305 | 99.5% | 96.7% |

**Table S2 – Draft contigs that mapped to the complete genome sequence of *A. acidoterrestris* DSM 3922^T^.** Data obtained using Minimap2. ‘_’ represents contigs that overlapped and were merged; ‘r’ represents the reverse contig sequence.

| **Sequence mapped** | **Mapped draft contigs** |
| --- | --- |
| **Chromosome** | AURB01000128r |
|  | AURB01000072r_AURB01000197r |
|  | AURB01000154r_AURB01000053 |
|  | AURB01000206r |
|  | AURB01000127 |
|  | AURB01000172r |
|  | AURB01000024_AURB01000069 |
|  | AURB01000004r_AURB01000001r |
|  | AURB01000132 |
|  | AURB01000148r |
|  | AURB01000066 |
|  | AURB01000014r |
|  | AURB01000177 |
|  | AURB01000038r |
|  | AURB01000041 |
|  | AURB01000033r |
|  | AURB01000090r |
|  | AURB01000031 |
|  | AURB01000003 |
|  | AURB01000096 |
|  | AURB01000202r |
|  | AURB01000017 |
|  | AURB01000175r |
|  | AURB01000054r_AURB01000136 |
|  | AURB01000078r |
|  | AURB01000043 |
|  | AURB01000147 |
|  | AURB01000191 |
|  | AURB01000184r |
|  | AURB01000146r |
|  | AURB01000062r |
|  | AURB01000151 |
|  | AURB01000201 |
|  | AURB01000190r_AURB01000121 |
|  | AURB01000173r |
|  | AURB01000164 |
|  | AURB01000174 |
|  | AURB01000163 |
|  | AURB01000140r |
|  | AURB01000176r |
|  | AURB01000089 |
|  | AURB01000048 |
|  | AURB01000018r |
|  | AURB01000006_ |
|  | AURB01000040r |
|  | AURB01000133r |
|  | AURB01000021 |
|  | AURB01000093r |
|  | AURB01000165r_AURB01000167 |
|  | AURB01000139r |
|  | AURB01000002 |
|  | AURB01000025 |
|  | AURB01000057r_AURB01000085 |
|  | AURB01000156 |
|  | AURB01000145r |
|  | AURB01000112r |
|  | AURB01000111 |
|  | AURB01000205r |
|  | AURB01000064r |
|  | AURB01000179 |
|  | AURB01000192r |
|  | AURB01000152r |
|  | AURB01000153 |
|  | AURB01000068 |
|  | AURB01000102r |
|  | AURB01000187r |
|  | AURB01000185r |
|  | AURB01000138 |
|  | AURB01000104r |
|  | AURB01000129r |
|  | AURB01000015r |
|  | AURB01000074r |
|  | AURB01000077_AURB01000168r |
|  | AURB01000080 |
|  | AURB01000034 |
|  | AURB01000009_AURB01000178r |
|  | AURB01000087 |
|  | AURB01000199 |
|  | AURB01000103r |
|  | AURB01000056r |
|  | AURB01000120r |
|  | AURB01000107r |
|  | AURB01000134 |
|  | AURB01000076_AURB01000119 |
|  | AURB01000189r |
|  | AURB01000071r |
|  | AURB01000169r |
|  | AURB01000101 |
|  | AURB01000079_AURB01000045r |
|  | AURB01000058 |
|  | AURB01000086r |
|  | AURB01000161 |
|  | AURB01000020r |
|  | AURB01000067 |
|  | AURB01000109 |
|  | AURB01000196r |
|  | AURB01000008r |
|  | AURB01000042r |
|  | AURB01000159 |
|  | AURB01000194r |
|  | AURB01000094r |
|  | AURB01000049 |
|  | AURB01000200r |
|  | AURB01000027r |
|  | AURB01000182 |
|  | AURB01000082r |
|  | AURB01000083r |
|  | AURB01000055r |
|  | AURB01000149_AURB01000124r_AURB01000091r |
|  | AURB01000198 |
|  | AURB01000037_AURB01000097r |
|  | AURB01000023r |
|  | AURB01000180_AURB01000016_AURB01000110r |
|  | AURB01000036_AURB01000115r |
|  | AURB01000035r |
|  | AURB01000188 |
|  | AURB01000193_AURB01000060r |
|  | AURB01000013r |
|  | AURB01000160r |
|  | AURB01000117r |
|  | AURB01000005 |
|  | AURB01000061r |
|  | AURB01000022 |
|  | AURB01000051r |
|  | AURB01000070r |
|  | AURB01000011r_AURB01000039_AURB01000092 |
|  | AURB01000059 |
|  | AURB01000183r |
|  | AURB01000029r |
|  | AURB01000126 |
|  | AURB01000141_AURB01000137 |
|  | AURB01000084 |
|  | AURB01000135r |
|  | AURB01000130 |
|  | AURB01000019r |
|  | AURB01000162 |
|  | AURB01000125 |
|  | AURB01000181 |
|  | AURB01000047 |
|  | AURB01000052 |
|  | AURB01000113 |
|  | AURB01000186r |
|  | AURB01000095 |
|  | AURB01000007 |
|  | AURB01000116r |
|  | AURB01000105 |
|  | AURB01000099r |
|  | AURB01000030 |
|  | AURB01000081 |
|  | AURB01000065r |
|  | AURB01000098r |
|  | AURB01000131 |
|  | AURB01000123r |
|  | AURB01000158 |
|  | AURB01000166_AURB01000144r |
|  | AURB01000075r |
|  | AURB01000171_AURB01000170r |
|  | AURB01000026r |
|  | AURB01000157r |
|  | AURB01000028 |
|  | AURB01000046r |
|  | AURB01000088r |
|  | AURB01000044r |
|  | AURB01000143r |
|  | AURB01000050r_AURB01000204r |
|  | AURB01000195r |
| **Plasmid** | AURB01000106_AURB01000150 |
|  | AURB01000155 |
|  | AURB01000100r |
|  | AURB01000108r_AURB01000203 |
|  | AURB01000032r |
|  | AURB01000207r |
|  | AURB01000073_AURB01000142_AURB01000118 |
|  | AURB01000114r_AURB01000063r_AURB01000010r |
|  | AURB01000012_AURB01000122r |

**Table S3 – Sequence annotations of former gaps present in the draft genome of *A. acidoterrestris* DSM 3922^T^.**

| **Sequence annotation** | **Nº of repetitions in the complete genome** |
| --- | --- |
| DDE-type integrase/transposase/recombinase | 10 |
| Integrase core domain-containing protein | 3 |
| IS110 family transposase | 7 |
| IS1380 family transposase | 18 |
| IS21 family transposase IstA | 3 |
| IS21-like element helper ATPase IstB | 4 |
| IS256 family transposase | 34 |
| IS3 family transposase | 9 |
| IS4 family transposase | 2 |
| IS66 family insertion sequence element accessory protein TnpB | 22 |
| IS66 family transposase | 13 |
| Transposase | 39 |
| **Total transposase/integrase annotations** | **161** |
| 16S rRNA | 12 |
| 23S rRNA | 12 |
| 5S rRNA | 13 |
| **Total ribosomal RNA annotations** | **37** |
| Ger(x)C family spore germination protein | 2 |
| Spore germination protein | 2 |
| **Total spore production related annotations** | **4** |
| AAA family ATPase | 9 |
| ABC transporter ATP-binding protein | 1 |
| ABC transporter permease | 3 |
| ABC transporter substrate-binding protein | 1 |
| DNA polymerase IV | 1 |
| DnaD domain protein | 1 |
| DUF5348 domain-containing protein | 7 |
| Group II intron reverse transcriptase/maturase | 2 |
| Helix-turn-helix domain-containing protein | 2 |
| Hypothetical protein | 12 |
| Urease accessory protein UreG | 1 |
| **Total annotations** | **241** |

**Table S4 – Number of coding sequences with protein in of *A. acidoterrestris* DSM 3922^T^ complete genome that were functionally annotated using KEGG and COG databases.**

| **Sequence annotated** | **# CDSs with protein** | **# CDSs with KO number** | **# CDSs with COG category** |
| --- | --- | --- | --- |
| **Chromosome** | 3,891 | 3,201 | 3,376 |
| **pDSM3922** | 108 | 81 | 64 |
| **Total** | 3,999 | 3,282 | 3,440 |

**Table S9 - Biosynthetic gene clusters identified in the complete genome of *A. acidoterrestris* DSM 3922^T^ using antiSMASH database.**

| **Region** | **Type** | **From (bp)** | **To**  **(bp)** | **Nº of genes in cluster** | | | | |
| --- | --- | --- | --- | --- | --- | --- | --- | --- |
|  |  |  |  | **Core genes** | **Additional biosynthetic genes** | **Transport-related genes** | **Regulatory genes** | **Other genes** |
| 1 | Saccharide | 541,114 | 584,727 | 3 | 7 | 2 | 3 | 25 |
| 2 | Saccharide | 592,442 | 615,076 | 2 | - | 1 | 1 | 18 |
| 3 | Saccharide | 679,658 | 697,960 | 2 | 1 | - | 3 | 10 |
| 4 | Saccharide | 1,039,944 | 1,086,184 | 3 | 7 | 2 | 2 | 28 |
| 5 | Saccharide | 1,351,332 | 1,384,694 | 10 | 4 | 1 | - | 22 |
| 6 | β-lactone | 1,445,407 | 1,475,288 | 2 | 7 | 2 | 3 | 9 |
| 7 | Saccharide | 1,769,446 | 1,797,037 | 3 | 3 | 5 | - | 19 |
| 8 | Saccharide | 1,927,489 | 1,951,804 | 2 | - | - | - | 18 |
| 9 | Fatty acids | 2,013,074 | 2,033,028 | 1 | 4 | - | - | 18 |
| 10 | Saccharide | 2,375,417 | 2,411,575 | 2 | 4 | - | - | 28 |
| 11 | Saccharide, Terpene | 2,624,868 | 2,669,920 | 3 | 9 | 1 | 5 | 31 |
| 12 | Saccharide | 2,679,075 | 2,717,240 | 3 | 8 | 4 | 1 | 20 |
| 13 | Fatty acids | 3,371,621 | 3,391,824 | 1 | 1 | 1 | 2 | 16 |
| 14 | Saccharide | 3,597,372 | 3,616,160 | 1 | 6 | 1 | 1 | 7 |
| 15 | Saccharide, Terpene, Fatty acids | 3,750,629 | 3,825,632 | 10 | 12 | 6 | 3 | 37 |
| 16 | NRPS-like | 3,902,900 | 3,946,547 | 1 | 7 | 5 | 4 | 17 |
